# Engaging Indigenous older adults with technology use to respond to health and well-being concerns and needs

**DOI:** 10.1177/08404704221103521

**Published:** 2022-06-07

**Authors:** Cari D. McIlduff, John Acharibasam, Victor Starr, Meghan Chapados

**Affiliations:** 1 7235 University of Saskatchewan, Saskatoon, Saskatchewan, Canada.; 2 12371 Star Blanket Cree Nation, Lebret, Saskatchewan, Canada.

## Abstract

Increased access to technology can promote independent living, stimulate cognitive functioning, relieve caregiver stress, improve telehealth access, increase overall well-being, and be used to share cultural resources such as Indigenous language applications. Many Indigenous older adults would like to learn more about technology and recognize the value of technology in supporting healthy ageing; however, as Morning Star Lodge has previously determined, accessibility and readiness were key factors in the use of this technology. Utilizing the guiding principles of the Model of Engaging Communities Collaboratively and the Ethical Engagement Training Module, Morning Star Lodge partnered with the Star Blanket Cree Nation to support the healthy lifestyle of six Indigenous older adults by increasing their access to and engagement with culturally safe technology solutions individual to their specific health and lifestyle needs. These co-researchers were provided with tablets, MiFis (mobile internet access), and learning workshops and were interviewed pre- and post-workshops to assess their comfort level with the device and information received. Additionally, these interviews assessed how the technology helped to address the health needs of the co-researchers. The findings demonstrated that the technology met the health needs of the older adults, particularly with the emergence of the COVID-19 pandemic and the need to stay connected to loved ones. The information gained through this work will support public health workers in responding to the needs of older Indigenous adults using technology to meet their health and well-being. There is also a significant need for pandemic preparedness work to be done with Indigenous communities and this work could inform this in part.

## Introduction

Increased access to technology can promote independent living, stimulate cognitive functioning, relieve caregiver stress, increase overall well-being, and be used to share cultural resources such as Indigenous language applications.^1–5^ Many Indigenous older adults would like to learn more about technology and recognize the value of technology in supporting healthy ageing; however, as Morning Star Lodge has previously concluded, accessibility and readiness were key factors in the use of this technology.^1^ Globally, access to technology and infrastructure, as well as awareness of what is available, can be limited. Barriers to access caused by infrastructure need to be addressed, especially in the context of COVID-19, which has ignited significant changes and restrictions in healthcare and physical distancing. With the emergence of the COVID-19 pandemic and the need to stay connected, access to culturally appropriate technology for remote Indigenous adults has become critical. Broadly, evidence suggests technology use among older adults in Canada increased due to the COVID-19 pandemic.^6^ But as Czaja observed, there is still the need to understand technology adoption and factors influencing technology uptake among subpopulations even if there is a general increase in technology use.^4^

Importantly, Czaja concluded, “... for the potential of technology to be realized for older adults, it is essential to understand trends in technology adoption and factors influencing technology uptake among older individuals.”^4^ This understanding is significant for health leadership in Saskatchewan as the province is expanding virtual healthcare services to improve access to healthcare.^7^ Limited access to technology among older adults is a major policy concern in Canada.^8^ Particularly, for Indigenous older adults living in remote communities, access to culturally safe technology still remains a challenge.^9,10^ This study is therefore timely and relevant to health leadership in Saskatchewan as it highlights some of the deeper socio-cultural factors that influence technology use among Indigenous older adults within Star Blanket Cree Nation.

This research involved collaborating with older Indigenous adults to determine what they want and need from technology to both support a healthy lifestyle, while increasing access to and engagement of culturally safe technology solutions. Scholars have shown the need to assess the distinct technology needs of older adults before engaging them with different types of technologies.^11,12^ Specifically, the researcher worked with six Indigenous older adults to identify technology needs through interviews and observation, where participants were provided with internet access and tablets (pre-loaded with individualized culturally safe and relevant applications [apps] and an unlimited data plan as required). Educational workshops were offered to support the use of the new devices and the language apps. Prior to engaging co-researchers, the researcher compared and contrasted the Model of Engaging Communities Collaboratively (MECC)^2^ and the Ethical Engagement Training Module (EETM)^3^ to ensure ethical engagement principles of research for Indigenous peoples in Treaty 4, Saskatchewan.

The research objectives of this work were to learn from Indigenous older adults about what they need, how competent they are/feel in using technology to meet these needs, and increase access to and engagement with technology specific to their overall well-being and healthcare. While the present climate of uncertainty weighs especially on Indigenous older adults about the safety of their health, the researcher aimed to increase their awareness of available resources and services that may help to alleviate any extra anxieties regarding their health. The research is also a pilot study of using the MECC and the EETM to support older Indigenous adults in addressing their health and well-being concerns and needs while also evaluating the processes of both these frameworks to further our understanding of working effectively and respectfully with Indigenous populations.

### Model of engaging communities collaboratively and the ethical engagement training module

The MECC and the EETM are models for engaging ethically and respectfully with Indigenous communities. The MECC was developed by Dr. McIlduff to inform the processes of implementing evidence-based practices with Indigenous populations in the Australian context. The EETM on the other hand was developed by Morning Star Lodge (an Indigenous community-based health research lab) in the Canadian context for engaging ethically and respectfully with Indigenous communities. Community engagement as used in this article means, building relationships with individuals and community members upon trust, cooperation, and mutual respect which extend beyond the life of a project.^13^

## Methods

First, MECC and the EETM were compared and contrasted to inform the work with Indigenous older adults in Saskatchewan. This knowledge was utilized to engage a Community Research Advisory Committee, recruit co-researchers, conduct pre- and post-interviews, as well as implement educational workshops. Indigenous community-based research methods were essential to this work. Observationally, data pertaining to the co-researchers’ competence to use technology was also collected through the workshops and involvement in the community allowed the researcher to observe familial interaction using various technologies. Pre-interviews focused on building relationships, such as getting to know the co-researcher, their health needs and experience, as well as confidence or curiosity around technology. After pre-interviews were completed, tablets were pre-loaded with individualized health and well-being apps according to each co-researchers specified health and well-being concerns. These tablets, along with a MiFi device with unlimited data for six months, were delivered to each co-researcher. Given the timing of the delivery, at the height of the second wave of COVID-19, the researcher was not able to give in-person support to assist with technology familiarity. This added an intergenerational dynamic to the project as children or grandchildren of the co-researchers were recruited to support their family members with the initial use of the technology. The first two educational workshops were conducted using Zoom and the last four workshops were conducted in person. Often, the children and grandchildren of the co-researchers still chose to participate in supporting their family members for the in-person workshops. The first two workshops reviewed the functionality of the tablet, including general functionality, volume, camera, zoom, security, and walking them through what apps they had on their tablets. The following four workshops were in person and therefore were more in depth, in which the co-researchers learnt how to use some of the apps available, how to add additional apps they wanted on the tablet and some even set up social media accounts to keep in contact with family and community. Post-interviews focused on confidence changes with using technology, future technology learning, their health, and their experience with the research process. These were open ended questions giving co-researchers the opportunity to express their views on the technology. Each interview lasted about an hour. Within the interviews, quantitative data was collected verbally using a Likert scale (see Table 2). The main purpose of the quantitative data was to measure how satisfied the co-researchers were with the entire research process.

### Ethics

This project received ethics approval from the Ethics Board at the University of Saskatchewan, Approval number 2292.

### Data analysis

The comparison of the similarities and differences amongst the guiding principles of the MECC and EETM was done with the support of the community research assistant to ensure cultural appropriateness of the comparison and further feedback. The qualitative results of the pre- and post-interviews were analyzed using an Indigenous data analysis method known as Nanâtawihowin Âcimowina Kika-Môsahkinikêhk Papiskîci-Itascikêwin Astâcikowin (NAKPA, Cree words meaning Medicine/Healing Stories, picked, sorted and stored). NAKPA data analysis is a process where, “a panel of experts, community members, participants, Elders, Knowledge Keepers, and the researchers are gathered together to do the collective data analysis.”^14^ Meaning the themes were co-deduced manually from the interview transcripts by the NAKPA panel. This data analysis approach aligns with the Community-Based Participatory Research methodology by directly involving the community in the analysis process. Hence, the data analysis was a collaborative process that involved the community. In this research, the panel consisted of researchers and Indigenous community members. The results were reviewed and approved by the community research assistant to ensure the results represented the co-researchers’ and community’s views. It is important to note that Indigenous community members were a part of the entire research process from its conception to the data analysis and knowledge mobilization. The quantitative data was also analyzed manually.

## Results

For the sake of this paper, it was determined that both MECC and EETM had similar considerations, but also complemented one another. The EETM named Cultural Safety, Self Determination, and Data Sovereignty overtly; however, the more prescriptive/practical nature of the MECC embeds these imperative concepts into what is expected of researchers throughout the process as can be seen in Table 1. The MECC could benefit from utilizing the OCAPⓇ, FAIR, and CARE principles, similarly to how the EETM utilized the OCAPⓇ principles.^15,16^ The EETM could benefit from more practical examples of how to attain ethical engagement similarly to what the MECC provides.

**Table 1.** Ethical engagement training module and model of engaging communities collaboratively comparison/alignment.

EETM	MECC
- Cultural safety	- Community identified concerns and solutions - Implementation - Dissemination approval
- Community-based partnerships - Communities as co-researchers	- Community identified concerns and solutions - Collaborative community consultation - Engagement of locals, leaders, and organizations - Identify cultural traditions, values, and beliefs - Implementation - Determine ecological fit and sustainability - Dissemination approval
- OCAP principles	- Implementation - Determine ecological fit and sustainability
- Traditional knowledge	- Community identified concerns and solutions - Collaborative community consultation - Identify cultural traditions, values, and beliefs
- Reciprocal learning	- Community identified concerns and solutions - Engagement of locals, leaders, and organizations - Identify cultural traditions, values, and beliefs - Implementation - Determine ecological fit and sustainability - Dissemination approval
- Integrated knowledge translation	- Implementation - Determine ecological fit and sustainability - Dissemination approval
- N/A	- Collaborative adaptation

## Demographics

The six co-researchers were between the ages of 55 and 81 (five females and one male). All but one of the co-researchers had multiple comorbidities. The one co-researcher who reported good health had significant concerns as a caregiver for other family members who had multiple comorbidities.

### Combining ethical engagement training module and model of engaging communities collaboratively for community engagement

The results showed that combing the two engagement models to facilitate the study ensured the research was conducted ethically. The co-researchers were asked about how they thought the research was done or if they would change how it was done. One co-researcher stated, *“I think it went really good. I really enjoyed it”* (co-researcher; CR 2). Another said, *“I thought it worked alright, lots to learn”*(CR 4)*.* A third co-researcher even identified the need for continued support after the research project formally ends. As she said, *“That will be something, you know, like you coming out again and show us more and stuff, I really liked it, how it went, you did good you know”* (CR 5).

This satisfaction was also reflected in the quantitative survey (Table 2) in which all questions were answered as agree or strongly agree to the good way in which research was done for each of the co-researchers. The questions ranged from, “I felt my concerns were heard and addressed by the researcher and workshops” to “I think the way the data was collected was done in a good way” and “I felt that this work was done in an ethical and culturally safe way.” Unfortunately, one co-researcher went on her spirit journey to the other side so was not included in the post data collection.

**Table 2.** Research process results.

Question	Strongly agree	Agree	Disagree	Strongly disagree
Overall, I am satisfied with how the researcher brought the project to our community	1	4		
I felt my concerns were heard by the researcher	3	2		
I felt my concerns were addressed by the researcher and the workshops	2	3		
I feel that we had enough involvement in the decision making	4	1		
I think the project fit with our needs well		5		
I think the workshops are a good fit for our community	5			
I think the person who taught the workshops was a good choice	2	3		
I think the people in the CRAC were a good choice	2	3		
I think the way the data was collected was done in a good way		5		
I felt the workshops were more community-needs led than researcher led	2	3		
I felt the way this work was done was ethical	1	4		
I felt the way this work was done was culturally safe	2	3		

### Pre-interview themes

As stated earlier, the pre-interviews were to assess the technology needs of the co-researchers. This information informed the type of apps that were pre-loaded on the tablets. The major themes that emerged from the pre-interviews were: history, health access, health, impact of the pandemic, technology use, and confidence. For the sake of this paper, only representative quotes were chosen in an effort to briefly and adequately showcase what was shared.

#### History

It emerged that historical factors were some of the factors that influenced the co-researchers’ technology needs. Meaning the co-researchers’ technology competency, how they felt about using technology, and the type of apps they wanted pre-loaded were informed by these factors. Specifically, some co-researchers spoke about how colonialism and the residential schools disrupted traditional family structures and support systems, thereby affecting their current health circumstances and technology needs. These factors therefore influenced technology competency and informed how the technology would be used to stay connected to family and the community at large. As one co-researcher commented on her family, *“[T]oday they’re all working and have their own jobs and have their own children to look after, they can’t be looking after us … Yeah, jobs and that take away people from families cause they have to work to survive … long ago they never had those, they never were they were self-efficient they lived off the land and lived off their own thing, today they have to live like they, they’re living in a society where they have to support themselves somehow and their families and even that having to work is taking away from being from family, their own kids”* (CR 1). Additionally, a second co-researcher noted, *“I lost my childhood, missed part of that circle, I was just busy looking after my siblings and family [in residential school]”* (CR 4).

#### Health access

Another important theme that emerged in the pre-interviews was that health access played a significant role in how the co-researchers would use the technology. We found that the difficulties in accessing western healthcare system and the need for aid/support informed how the co-researchers would use the technology as well as the type of apps they wanted to meet their health needs. With most of the co-researchers being older adults, there was a strong need for social and healthcare from family members. Hence, they stated that although the technology would help them stay connected with their loved ones, having a family member living within the same community or physically present was also preferred. As one co-researcher stated when talking about her daughter, *“Yeah, she lives right in the town here so it’s easy for her, for me if I needed homecare I would have a hard time out here, we have no funding whatsoever to hire homecare, people from out of our own community to help us”* (CR 1). Another identified health access as a significant concern, particularly where aid/support was involved, *“I have 3 other people helping now it used to be just me and I told them I was feeling my health”* (CR 3). They also spoke of the importance of ancestral healing practices, *“Praying I guess and spiritual, smudging trying to stay positive when things are going wrong”* (CR 4).

#### Health

Another major theme that emerged from the pre-interviews was that individual co-researcher’s health concerns and the health concerns of loved ones or family would influence technology use and the type of apps they wanted on their tablets. Commenting on her current health concerns, a co-researcher explained, *“I kind of think I’m getting arthritis ‘cause I’m having a lot of joint pain in my knees and it’s hard to go up and down the stairs a bunch”* (CR 2). She went further to state that, *“I notice the side where I have this bunion or whatever it’s called, it hurts my foot underneath like there’s a bone there”* (CR 2).

A second co-researcher also spoke of her family’s health as it affects her own life by stating she is *“Not a diabetic, but my youngest son is, he also has high blood pressure, I worry about him”* (CR 4). She went further to explain that, *“There’s a lot of respiratory problems in my family on my mother’s side—A lot of them had to be on oxygen and things like that”* (CR 4). One co-researcher spoke of the experience of health changes, *“My health being bad now, it’s hard to do those things I used to like”* (CR 6).

#### Impact of the pandemic

Also, the pre-interviews showed the COVID-19 pandemic influenced the co-researchers use of the technology and the type of apps they wanted installed. With the emergence of the pandemic and the institution of social distancing regulations, the need to stay connected to loved ones and family was important for most co-researchers. This therefore informed how they would use the technology. Family plays a very important role within Indigenous communities, the isolation that came with the COVID-19 pandemic was therefore a health need that the co-researchers thought could be solved using the technology. As one co-researcher noted, *“I’m not seeing family because of the pandemic, it’s very isolated times”* (CR 5).

#### Technology use and confidence

On co-researchers’ experience with technology and confidence in using the new technology, the pre-interviews showed their technology needs were influenced by language and culture, communication and staying connected and hesitancy and risk. Hence, communication apps and staying connected to the community were important to some of the co-researchers. As one co-researcher noted, *“Sometimes we go on my husband’s phone, we do facetime.”* She went on to state *“I would like to learn more about zoom to attend community meetings”* (CR 3)*.*

Again, culture and language also came out as strong themes that influenced the co-researchers' technology needs. Some co-researchers talked about the need for Indigenous language apps and to reconnect with cultural teachings. Therefore they wanted the technology to help facilitate these cultural learning processes. A co-researcher, for example, commented, *“I actually took a few sessions of Cree on my phone, I enjoy learning about history, history of Indigenous peoples”* (CR 5). Another co-researcher stated, *“I use it to pray in the morning and pray in the night when it comes on my phone”* (CR 2).

However, some co-researchers expressed concerns about the risk associated with technology use and the fact that they did not know much about the technology. Co-researchers spoke of the frequency of technology use, hesitancy in using technology and the risks associated with technology use. A co-researcher stated that, *“Security is a concern with all the scammers out there”* (CR 4). A second co-researcher also observed, *“I’m just finding out about it [technology], I’m not really that good to tell you the truth”* (CR 3). A third noted *“Not many Elders are interested in these things—like technology”* (CR 6).

### Post-interview themes

The major themes that emerged in the post-interviews were health, confidence, education, and the generational gap. For the sake of this paper, only representative quotes were chosen in an effort to briefly and adequately highlight what was shared.

#### Health

As observed in the pre-interviews, when speaking about their health, the co-researchers included their family’s health as it also impacts the information they were seeking while using technology. Health influenced how the technology was used and the type of information the co-researchers used. As one co-researcher noted, *“My nephew, he has to go for a kidney transplant, he is trying to find a donor, they are testing, gonna get tested to donate to him. They say a live donor would be better”* (CR 2). Another co-researcher observed, *“I’m getting such short memory now, so things go in but don’t stay in … like you said, I might have to, you know, start writing stuff down in order to try and do stuff”* (CR 4).

#### Confidence

Following the workshops, while co-researchers still spoke to their hesitance, lack of confidence, and security concerns, their familiarity and use of technology had greatly increased. We found the educational workshops helped enhance the co-researchers’ confidence in using the new technology. One co-researcher stated, *“I’m comfortable with it, I just got to know a little bit more, I get frustrated”* (CR 5). A second noted, *“Yeah, I’m getting there [more confident] … when I use them”* (CR 3). However, a third co-researcher admitted her confidence had increased but the rate at which new technologies and apps were being developed posed a threat to her learning about technology. By observing, *“I’m not too well advanced in that, in the technology. Once I learn something then there’s something else new coming up. There’s something new every year, yeah like you know … I guess if I just you know, learn the basics and whatever I guess I’d be just fine with me”* (CR 4).

We observed that although confidence in technology use improved, there was still some hesitancy on the use of technology among co-researchers. One co-researcher noted, *“Oh my goodness! I don’t wanna get too dependent on technology”* (CR 5). Another co-researcher stated, *“Sort of comfortable, I’m very careful about that. Yeah, I don’t go on-line like for Amazon or anything like that, I don’t purchase”* (CR 4).

She went further to state that *“Kind of scary and then you … I’m listening to the news like every day, like what else can I do, but you know, listen and hear about you know scammers and whatever”* (CR 4).

Further enquiries revealed some older adults within the community were victims of internet scams. Perhaps this explains some of the hesitancy around technology use among the co-researchers. Irrespective of this unfortunate experience, the post-interviews showed the co-researchers’ knowledge of the interconnectedness of many technologies grew significantly as they became more adventurous with the support of the workshops. As one co-researcher stated, *“My e-mails are connected to both my cell and my personal computer”* (CR 5). A second co-researcher went further to show how she has been able to use the tablets for on-line banking. She noted, *“I do my on-line banking on my phone. I don’t do it on my personal computer. It’s more convenient on my phone”* (CR 2).

Again, others spoke openly of their new found confidence and understanding and trouble shooting. A co-researcher, for example, observed, *“I probably will, you know, help me you know doing things on my tablet, I probably will get a different phone with those kinds of apps and whatever on them”* (CR 4). Another spoke about the internet connectivity being slow. She stated, *“It’s the wifi, I even bought an extra cable and even that is not connecting”* (CR 5). Some of the co-researchers even added more apps to the technology. One co-researcher, for example, stated, *“I don’t understand like Dropbox. Like how, like my files how to store them, I just store them wherever. I need to kind of look for them after”* (CR 5).

The post-interviews also showed some of the co-researchers used technology to access healthcare. Access to healthcare services remains a major challenge for older Indigenous adults living in remote communities. It was therefore important to find out that the technology was being used to access healthcare. Some of the co-researchers spoke of their (or their family’s) experience using telehealth. As one co-researcher stated, *“Actually, my doctor phones me when I can’t go in. So she set up a certain time that she would call between eleven and twelve and asked me all kinds of questions and stuff like that and yeah”* (CR 6). She further observed, *“He usually calls and checks up. He’s had his family doctor for over 20 years. So he’s really good with him. The doctor calls him, even on Saturday to see how he’s doing. I like that part about him”* (CR 5).

#### Education

Another important theme that emerged from the post-interviews was around education. When co-researchers spoke of their own education around technology, they spoke of what they had already been learning and how they were sharing this knowledge, and what they would like to learn in the future. One co-researcher said, *“So you know it’s I just watch and stuff like that and then I forget about it, eh, for a while. And then I go back and you know try and figure out stuff and things like that. So, I mean it comes to me eventually”* (CR 4). Another co-researcher went further to talk about how technology could help with daily chores. As she noted, *“I got a vacuum, robot vacuum cleaner. I don’t really know how to use the damn thing”* (CR 3). Some co-researchers have even learnt to sync their accounts into a single account. Based on this a co-researcher commented, *“All my devices are connected to my Bell account”* (CR 5). We also found some of the co-researchers were willing to help their partners with the use of technology. One co-researcher spoke about her partner said, *“So I don’t know what’s happening with his phone or if he needs a different phone, I always tell him he should get a bank, get your bank apps on your phone. But I don’t know if he’s able to do that”* (CR 5). Additionally, they talked about which technology devices they preferred based on different functions. One co-researcher stated, *“I would much rather use my computer, ‘cause it’s got the bigger screen. But yeah, if I have to, if I’m on the road, it’s really convenient with my cell phone ‘cause I can get Zoom and everything and my e-mails on my phone”* (CR 6 ). When they spoke of their technology literacy, the ranges of what they could use had really expanded, *“I don’t know anything on my phone except text and phone and play games and Facebook.”* *“I used to have games and then I deleted them, ‘cause I didn’t want to use up my gigabytes or whatever.”* *“The apps I do have right now, I like to get onto the iTunes - I have that and I have my banking app.”*

Many expressed interest in further learning around different technologies both for themselves and their fellow Elders, *“If they [Elders] knew about it [technology], maybe they would, but I think we’re just kind of like in isolation right now. ”* *“I just don’t know how, my printer … I can’t get connected to my computer.”* *“And like, even now you know, like more people get, like this workshop.”* *“ We need to make this available for more Elders to come out. ”*

#### Generational gap

A few of the co-researchers spoke of the generational gap created by technology, particularly around communication of current events occurring in their own communities. A co-researcher commenting on generational gap in technology use said, *“And then when something comes up and stuff like that, then they say—oh you weren’t you know, notified—and stuff like that, like a lot of them, you know, don’t have access, like I said, you know we’re learning this tablet and that and Facebook and that, eh, and that I’m sure a lot of them have phones, you know, and they can get all this message and whatever if they had Facebook on their phones”* (CR 3).

## Discussion

Community engagement plays a significant role in Indigenous research.^17,18^ Combining the EETM and MECC models ensured effective community engagement and collaboration in the research process. The strengths of both models ensured a continuous community involvement throughout the entire research process. Importantly, engaging the community in every stage of the research, including allowing the co-researchers to decide the type of apps to be loaded into the technology ensured the co-researchers were respected as equal partners in the research. Besides this, the community was also involved in the data analysis stage, reviewed and accepted the results before this article was submitted for publication. Again, this enhanced respect and trust which were very important for technology education during this research. Given the unfortunate experience of one of the co-researchers falling victim to internet scams, trust was very important in building the co-researchers’ confidence in using the technology. Also, the involvement of young family members in the technology education process helped build trust in the technology. The COVID-19 pandemic created an opportunity to engage young family members in teaching co-researchers to use the technology. Fortunately, most of the things taught by the researcher and the young family members were similar. Hence, further creating trust among the co-researchers in the technology and research process. Trust is therefore crucial in building older adults’ confidence in technology use and involving family members can help build this trust. This can be achieved through effective collaboration methods as shown by Table 2 assessing the co-researchers' satisfaction with the research process.

### Health access

When informed by Indigenous culture, technology can improve Indigenous older adults’ access to healthcare.^19^ The health of the co-researchers and their family members played a significant role in how they engaged with the technology. This was seen in the type of apps and information the co-researchers used. Most of these were centred around their health and the health of their family as well as loved ones. Tailoring technology to meet the specific health needs of older adults and their families can therefore enhance access and build confidence in technology use. Meaning it is also important to consider the families of older adults when engaging them in technology since family plays an important cultural role within Indigenous communities. The health and well-being of family members is as important as the co-researchers' own health and well-being. Additionally, it is important to note that Indigenous peoples view health as being holistic.^20^ Hence, the technology was also used to meet spiritual health needs including prayers. Basing technology education on a holistic concept of health is therefore important when engaging Indigenous older adults in technology use.

### Culture

Again, culture is very important when engaging Indigenous older adults in technology use.^19,21^ As Choukou, Maddahi, Polyvyana, and Monnin observed, respect for Indigenous culture is key to Indigenous older adults’ acceptance of technology.^19^ Hence, incorporating Indigenous culture including Indigenous languages, songs, stories, ceremonies, and prayers improved the co-researchers' confidence in using the technology. Culture defined how the co-researchers used the technology and the type of apps and information they searched for. Specifically, some of the co-researchers reported how the technology helped them reconnect with their culture including using the technology for praying and learning their language.

### Technology education

Centring technology education around Indigenous peoples' cultures and traditions improves access.^21^ This study also shows that involving family in technology education improves confidence in technology use among Indigenous older adults. Additionally, some of the co-researchers emphasized the need for Elders to lead technology education within their community. Unlike Western learning systems, learning within most Indigenous communities is integrated into the fabric of both the family and the community as a whole.^21,22^

### Confidence in technology use

While the co-researchers continued to speak of their low confidence in using technology, they also expressed many additional applications and ways they were using their devices which showed they were gaining confidence, potentially even more than they realized. A prime example was co-researchers speaking of gigabytes and e-mail access on phones which were not common knowledge for them previously. They also learnt about the differences between LTE and wifi which some already had set up in their homes, but were not utilizing on their devices. The co-researchers showed improved knowledge around health services and information which supported their health and well-being needs and those of their family members. They also showed interest in social media platforms to further address their concerns and the communication gap due to isolation that they were experiencing with events going on in their community and their families. Further, all the co-researchers wanted to continue on this learning journey to learn more about technology.

### Opportunity for bridging the communication gap

Given the significant interest shown by the co-researchers and their motivation to share their new skills and this work with other older adults, this work could set a precedent for how other researchers can support Indigenous communities, nationally and globally to empower older adults in both bridging the generational communication gap often mentioned as a concern and addressing health access in this and future crises or pandemics. The audience/user groups who could benefit from the knowledge produced by this research include Indigenous older adults, Indigenous communities across Canada and globally, retirement homes/residences, homecare professionals, and other professionals/researchers working with these communities.

Furthermore, user-needs research is foundational to technology uptake. This research was directly responsive to the user-needs which required flexibility and innovative solutions to fit the unique needs of this population. Limitations of this work include a small sample size; however, this small sample size also allowed for more individualized support and solutions for each co-researcher and more rich data.

Also of note, while working with the tablets allowed the co-researchers to explore new technology and build confidence, it was also recognized that potentially a more effective way of building this confidence for a greater population would have been to utilize the technology that they already had been engaging with and build their confidence on these first before introducing a new technology. Given the individualized workshops, this certainly happened organically while the researchers were in their homes, but the money spent on the tablets could have been utilized to offer more workshops to more co-researchers.

## Conclusion

Given the need during the COVID-19 pandemic to continue to stay semi-isolated due to older Indigenous adults being susceptible to the virus, the information gained through this work has the capacity to support other researchers and public health workers to support Indigenous communities in Canada and globally in using technology to respond to older Indigenous adults’ health and well-being. There is also significant need for pandemic preparedness work to be done with Indigenous communities and this work could inform part of that preparedness, particularly for the older adult populations.

The MECC processes and EETM principles have been shown here to be effective and culturally appropriate as seen through both qualitative and quantitative responses with Indigenous participants in Canada, furthering the importance of ensuring research and work with Indigenous peoples must take a more consultative, collaborative and community-led approach across all fields of research and programming.
